# Prevalence and factors associated with pulmonary hypertension in patients undergoing maintenance hemodialysis: a systematic review and meta-analysis

**DOI:** 10.3389/fmed.2026.1889032

**Published:** 2026-09-28

**Authors:** Hong-wei Yang, Juan Wang, Hui Ji, Lin Gui, Yan Zhao, Zhong-jian Li, Ling-mei Ruan, Bing-hua Lan, Xiao-qian Wu

**Affiliations:** 1 College of Nursing Dali University, Dali City, Yunnan Province, China; 2 The Department of Nursing, Affiliated Hospital of Panzhihua University, Panzhihua City, Sichuan Province, China; 3 College of Health and Wellness, Panzhihua University, Panzhihua City, Sichuan Province, China

**Keywords:** maintenance hemodialysis, systematic review and meta-analysis, prevalence, pulmonary hypertension, related factors

## Abstract

**Background:**

Maintenance hemodialysis (MHD) is a primary treatment for end-stage kidney disease (ESKD). Patients on long-term MHD frequently develop cardiovascular complications, with pulmonary hypertension (PH) prevalence reported as high as 47%. Due to small sample sizes and inconsistent findings, associated factors for PH in MHD patients remain unclear.

**Objective:**

To evaluate the prevalence and associated factors of PH in MHD patients.

**Methods:**

We systematically searched literature on PH risk factors in MHD patients across PubMed, Web of Science, CINAHL, Cochrane Library, Embase, CNKI, CBM, Wanfang, and VIP (inception to April 20, 2026). Two reviewers independently screened and extracted data. Data were synthesized using Stata 18.0.

**Results:**

Thirty-nine studies comprising 6,633 subjects were included, of whom 3,930 were males and 2,703 were females. The pooled PH prevalence was 37% (95% CI: 0.32–0.42). Multiple factors were significantly associated with PH (*P* < 0.05). Significant associated factors included age ≥ 60 years (OR = 1.03, 95% CI: 1.02–1.05), vascular access modalities (OR = 7.84, 95% CI: 4.98–12.33), hs-CRP ≥ 6.0 mg/L (OR = 3.57, 95% CI: 2.41–5.30), interdialytic weight gain (continuous: OR = 18.39; binary classification: OR = 20.51), serous cavity effusion (OR = 3.03, 95% CI: 1.84–4.97), elevated NT-proBNP (OR = 1.07, 95% CI: 1.02–1.12), BNP increased (OR = 1.77, 95% CI: 1.06–2.97), E/A (OR = 3.00, 95% CI: 1.89–4.75), enlarged LAD (OR = 1.17, 95% CI: 1.09–1.25), enlarged LVEDD (OR = 1.11, 95% CI: 1.05–1.17), Increased LVPWTd (OR = 1.72, 95% CI: 1.31–2.26), enlarged RAD (OR = 1.17, 95% CI: 1.12–1.22), and valvular regurgitation (OR = 3.83, 95% CI: 2.39–6.13). Reduced LVEF (OR = 0.92, 95% CI: 0.87–0.97) was inversely associated with PH.

**Conclusion:**

The high PH prevalence in MHD patients, influenced by diverse factors, calls for enhanced clinical vigilance. Management should focus on early identification of high-risk patients, strengthened volume management, inflammation control, and cardiac monitoring. This approach is essential to lower PH risk and improve cardiovascular prognosis.

**Systematic Review Registration:**

https://www.crd.york.ac.uk/PROSPERO/view/CRD420261378052.

## Introduction

Chronic kidney disease (CKD) represents a major global public health challenge. According to the WHO, it affects approximately 674 million individuals, accounting for about 9% of the world's population (1). Pulmonary hypertension (PH), a significant cardiovascular complication of CKD, is primarily characterized by a sustained elevation in pulmonary arterial pressure. According to the 2015 European Society of Cardiology/European Respiratory Society (ESC/ERS) guidelines, PH was defined as a peak tricuspid regurgitation velocity (TRV) ≥2.8 m/s and estimated pulmonary artery systolic pressure (PASP) >35 mmHg (2). The echocardiographic prevalence of PH ranges from 21% to 27% in patients with chronic kidney disease (CKD) and reaches up to 47% in those with end-stage kidney disease (ESKD) (3). Moreover, studies (4, 5) have confirmed that PH is an independent risk factor for mortality in the Maintenance hemodialysis (MHD) population.

The pathogenesis of PH in patients undergoing MHD is complex and not fully elucidated. It is thought to involve multiple factors, including elevated pulmonary capillary wedge pressure (PCWP), left ventricular dysfunction, and chronic fluid overload (6). While a prior meta-analysis by Lin et al. (7) evaluated predictors of PH in patients with CKD, that study encompassed patients across various CKD stages, including non-dialyzed and pre-dialysis ESKD patients, without focusing specifically on the MHD subgroup. Importantly, due to the long-term effects of dialysis therapy, MHD patients differ from non-dialyzed CKD patients in terms of hemodynamic status, inflammatory response, and metabolic disturbances, which may uniquely influence the development and progression of PH (8). Studies (9, 10) have shown that dialysis vintage, left ventricular hypertrophy, and left ventricular ejection fraction are factors associated with PH in MHD patients, yet their impact remains inconsistent across studies. Existing studies exhibit significant heterogeneity in sample size, study design, definitions of outcome measures, and reported results. This heterogeneity has led to unclear identification of risk factors for PH in MHD patients, with some factors remaining insufficiently verified and a lack of systematic integration of evidence. Therefore, this study aimed to systematically search the relevant domestic and international literature to comprehensively evaluate the associated factors for PH in MHD patients. Our goal is to provide an evidence-based foundation for the early identification of high-risk individuals and the development of individualized intervention strategies, thereby improving cardiovascular prognosis and reducing mortality in this population.

## Methods

This review was conducted and reported in accordance with the PRISMA 2020 guidelines and the PRISMA 2020 checklist (Supplementary File 1). The protocol was prospectively registered in the International Prospective Register of Systematic Reviews (PROSPERO) under registration number CRD420261378052.

### Inclusion and exclusion criteria

#### Inclusion criteria

① Age ≥ 18 years old; ② Patients received regular maintenance hemodialysis; ③ The study reported at least one risk factor related to the target outcome; (4) provide original data (OR) of the risk association effect, OR provide data that can be calculated as OR; ⑤ The types of studies were cohort study, case-control study, or cross-sectional study; ⑥ Pulmonary artery systolic pressure was measured by Doppler ultrasound, and PASP was > 35 mmHg.

#### Exclusion criteria

① repeated publication, unavailability of full text, incomplete data, or inability to effectively extract and transform studies; ② Non-Chinese or non-English publications; ③ Reviews, conference abstracts, conference papers, guidelines, expert consensus, comments, and animal experiments; ④ The research topic or content is not consistent with the objective of this analysis.

### Literature search strategy

We conducted a systematic search in PubMed, Embase, Web of Science, CINAHL, Cochrane Library, CNKI, CBM, Wanfang, and VIP databases, covering all records from the establishment of the databases until April 20, 2026. The search was carried out using a combination of subject terms and free words. For Chinese, the search terms were (renal dialysis OR dialysis OR renal substitution therapy OR renal substitution OR hemodialysis OR hemodialysis filtration OR hemofiltration OR extracorporeal dialysis) AND (pulmonary hypertension) AND (influencing factors OR related factors OR risk factor OR predictive factor). For English, taking the Cochrane Library as an example, see Table 1. We also traced relevant references to identify potential studies that met the inclusion criteria. And the search formula was adjusted according to the specific circumstances of each database. The detailed search strategy can be found in Supplementary File 2.

**Table 1:** Cochrane library search strategies.

Search set	Search terms
#1	MeSH descriptor: [Renal Dialysis] explode all trees
#2	(Renal Dialysis):ti,ab,kw OR (Dialyses, Renal):ti,ab,kw OR (Renal Dialyses):ti,ab,kw OR (Dialysis, Renal):ti,ab,kw OR (Hemodialysis):ti,ab,kw
#3	(Hemodialyses):ti,ab,kw OR (Dialysis, Extracorporeal):ti,ab,kw OR (Dialyses, Extracorporeal):ti,ab,kw OR (Extracorporeal Dialyses):ti,ab,kw OR (Extracorporeal Dialysis):ti,ab,kw
#4	MeSH descriptor: [Pulmonary Arterial Hypertension] explode all trees
#5	(Pulmonary Hypertension):ti,ab,kw OR (Hypertension, Pulmonary):ti,ab,kw
#6	#1 OR #2 OR #3
#7	#4 OR #5
#8	#6 AND #7

### Literature screening and data extraction

The search results from all databases were imported into the reference management software NoteExpress (version 4.3.0; Aegean Software Corp., Beijing, China) (11) to facilitate deduplication and screening. Two reviewers (WXQ and LZJ) independently assessed study eligibility based on titles and abstracts, using predefined inclusion and exclusion criteria. Studies deemed potentially relevant proceeded to full-text review, which was also conducted independently by the same two reviewers. In cases of disagreement at any stage of screening, a consensus was reached through consultation with a third reviewer (YHW). Subsequently, data from the final set of included studies were extracted independently by the two reviewers (WYQ and LZJ) into a pre-designed Microsoft Excel (version 2024, Microsoft Corp., Redmond, WA, USA) (12). The extracted data comprised: first author, year of publication, country, study design, dialysis vintage, PH diagnostic criteria, sample size, and investigated risk factors, along with other pertinent information.

### Evaluation of literature quality

The methodological quality of the included studies was independently assessed by two reviewers (LBH and RLM). Any disagreements in assessment were resolved through discussion with a third reviewer (YHW) until consensus was reached. For cohort and case-control studies, quality was appraised using the Newcastle-Ottawa Scale (NOS) (13), which evaluates studies across three domains: selection of study groups, comparability of groups, and ascertainment of either the outcome (cohort studies) or exposure (case-control studies). The total NOS score ranges from 0 to 9, with studies scoring ≥ 7, 4-6, and < 4 considered high-, moderate-, and low-quality, respectively. For cross-sectional studies, quality was assessed using the JBI Critical Appraisal Checklist for Analytical Cross-Sectional Studies (14). This checklist comprises 8 items, each scored as 1 (“yes”) or 0 (“no”/“unclear”/“not applicable”). The total score ranges from 0 to 8. According to the classification proposed by Sun et al. (15), scores of 7–8, 5–6, and 0–4 indicate high, moderate, and low quality, respectively.

### Quality of evidence

The GRADE (Grading of Recommendations Assessment, Development and Evaluation) system (16) was employed to grade the quality of evidence for each risk factor. The system conducts a comprehensive evaluation across five dimensions: risk of bias, study heterogeneity, indirectness, imprecision, and publication bias, classifying the evidence quality into four levels: high, moderate, low, and very low. The results are presented in a summary of findings table.

### Statistical analysis

This meta-analysis was performed using Stata software (version 18.0). The pooled effect sizes are presented as odds ratios (ORs) with 95% confidence intervals (CIs). When studies reported both unadjusted and adjusted ORs, the latter were preferentially extracted; otherwise, unadjusted ORs were used. The adjustment status (unadjusted/adjusted/adjusted without specifying confounders) and the specific confounders adjusted for each study were recorded in detail. In addition, a sensitivity analysis was performed by the leave-one-out approach in this study to assess the robustness of the pooled results. For studies that reported only the logistic regression coefficient (*β*) and its standard error (SE), the corresponding OR and 95% CI were derived using the inverse natural log transformation: OR = exp (*β*), 95% CI = exp (*β* ± 1.96 × SE) (17). The internal consistency of the extracted data was verified by cross-checking the calculated ORs and the Wald *χ*^2^ statistic [*χ*^2^ = (*β*/SE)^2^], and studies with obvious transcription errors were excluded. Heterogeneity among studies was quantitatively assessed using the Q-test and the *I*^2^ statistic. A fixed-effects model was employed when heterogeneity was deemed acceptable (*P* > 0.1 for the Q-test and *I*^2^ ≤ 50%). Otherwise, a random-effects model was used. For risk factors with high heterogeneity (*I*^2^ ≥ 70%), potential sources were explored via leave-one-out sensitivity analysis or meta-regression. Publication bias was evaluated using Egger's test for risk factors that were examined in ten or more studies.

## Results

### Results of literature search

The initial search yielded 1,117 records. After removing 156 duplicates, 961 unique records remained for screening. Based on the title and abstract, 855 records were excluded. The full texts of the remaining 106 articles were assessed for eligibility, leading to the exclusion of 71 articles for the following reasons: non-conforming study population (*n* = 4), review article (*n* = 4), incomplete data/publication (*n* = 5), conference abstract (*n* = 3), non-conforming study focus (*n* = 50), full text unavailable (*n* = 4), and overlapping cohort (*n* = 1). An additional 4 studies were identified through manual searches of reference lists. Ultimately, 39 articles met al.l inclusion criteria and were included in the meta-analysis. The study selection process is detailed in the PRISMA flow diagram (Figure 1).

**Figure 1:**
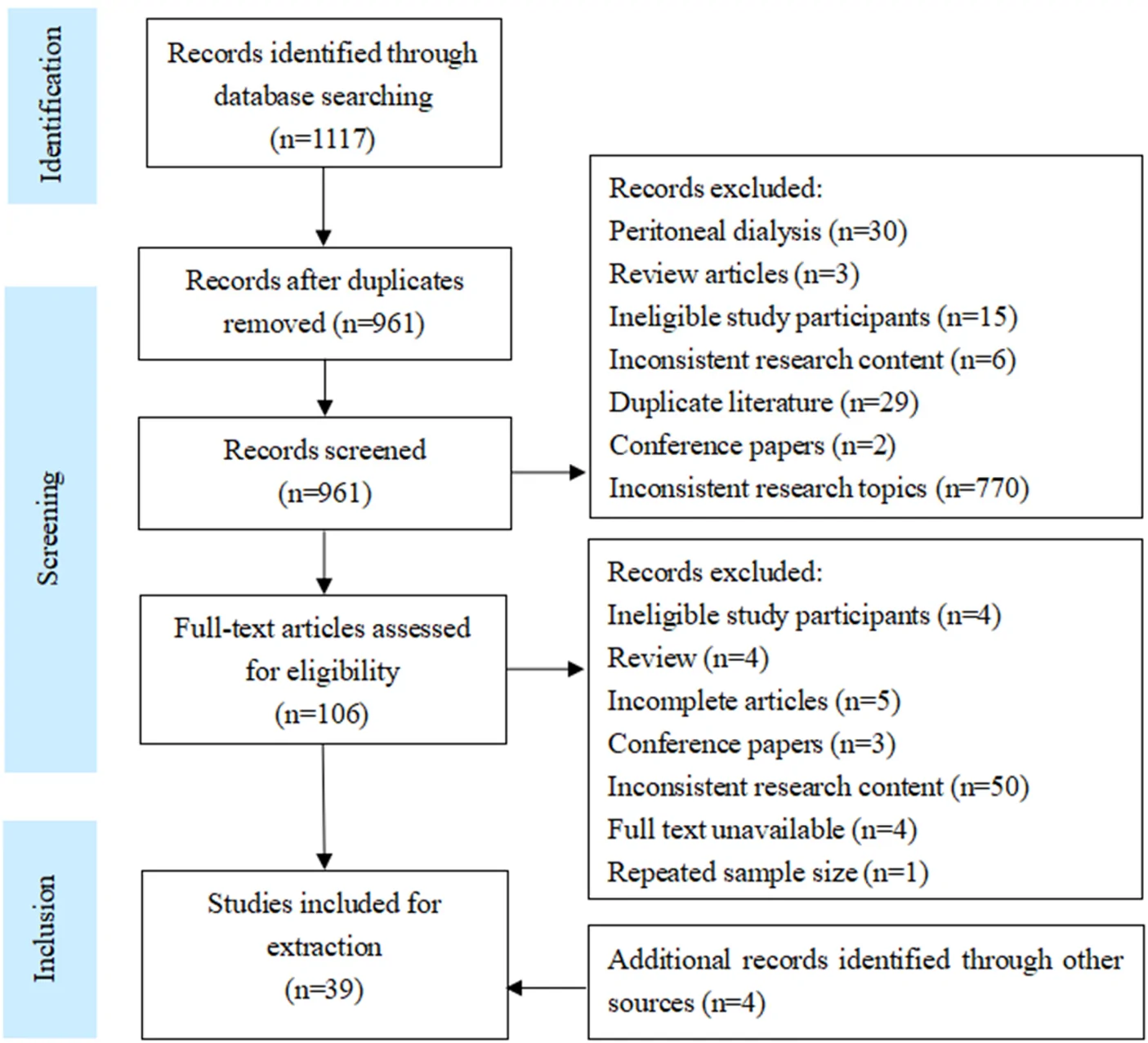
Flow chart depicting the literature screening process.

### General characteristics and quality evaluation of the included studies

A total of 39 studies were finally included, with publication years ranging from 2010 to 2026. Most studies (*n* = 34) were conducted in China, with the remaining 5 from the United States (*n* = 1), Italy (*n* = 1), Turkey (*n* = 1), the Republic of Congo (*n* = 1), and Somalia (*n* = 1). Among the included studies, 23 were cross-sectional studies, 12 were retrospective studies, and 4 were prospective studies. The sample size ranged from 33 to 834, with a total sample size of 6,633 cases, of whom 3,930 (59.2%) were males, and 2,703 (40.8%) were females. For effect measures, 36 studies (92.3%) reported only unadjusted odds ratios (ORs), while 3 studies (7.7%) reported adjusted ORs. Among the latter, one study (Hsieh et al.) explicitly adjusted for diabetes, coronary heart disease, smoking, and ejection fraction, whereas the other two studies (Ding et al. and Engole et al.) reported adjusted results without specifying the confounding variables that were adjusted for. Detailed characteristics and quality assessment results of the included studies are summarized in Table 2.

**Table 2:** Basic characteristics and quality evaluation of the included literature (*n* = 39).

Author	Publish year	Country	Research type	Duration of dialysis	Duration of dialysis per week	Diagnostic criteria for PH	Sample size (cases)		Prevalence PH (%)	Risk factors	Quality score
							Non- PH	PH			
Guo et al. (18)	2026	China	Retrospective study	≥ 3 months	3×/week, 4 h/session	PASP > 35 mmHg	93	66	41.5%	①②③	7
Zhou et al. (19)	2026	China	Cross-sectional study	≥ 3 months	–	PASP > 35 mmHg	757	77	9.2%	④	8
Zhang et al. (20)	2025	China	Cross-sectional study	≥ 3 months	3×/week, 4 h/session	PASP > 35 mmHg	219	30	12.0%	④⑤⑥	8
Dai et al. (21)	2024	China	Retrospective study	≥ 3 months	–	PASP > 35 mmHg	180	125	41.0%	④⑦⑧⑨⑩⑪⑫	7
Xu et al. (22)	2023	China	Retrospective study	≥ 6 months	3×/week, 4 h/session	PASP > 35 mmHg	206	129	38.5%	⑥⑩	7
He et al. (23)	2023	China	Retrospective study	≥ 6 months	–	PASP > 35 mmHg	49	31	38.8%	④	6
Li (24)	2023	China	Cross-sectional study	≥ 3 months	–	PASP > 35 mmHg	338	194	36.5%	⑥	8
Alici et al. (25)	2022	Somalia	Retrospective study	≥ 3 months	–	PASP > 35 mmHg	70	73	51.0%	⑨⑪⑫	6
Ding et al. (26)	2022	China	Prospective cohort study	–	–	PASP > 35 mmHg	95	61	39.1%	⑫⑯	9
Gao et al. (27)	2021	China	Cross-sectional study	≥ 3 months	–	PASP > 35 mmHg	22	11	33.3%	⑰	5
Shen et al. (28)	2021	China	Retrospective cohort study	≥ 3 months	3×/week, 4 h/session	PASP > 35 mmHg	95	124	56.6%	⑩	7
Tong et al. (29)	2020	China	Prospective study	≥ 6 months	–	PASP > 35 mmHg	74	53	41.7%	②⑬⑱⑲	7
Xu (30)	2020	China	Cross-sectional study	≥ 3 months	–	PASP > 35 mmHg	136	100	42.4%	②⑬	8
Engole et al. (31)	2020	Republic of the Congo	Cross-sectional study	≥ 6 months	–	PASP > 35 mmHg	60	25	29.4%	㉔	7
Zhang et al. (32)	2020	China	Cross-sectional study	–	–	PASP > 35 mmHg	172	111	39.2%	④⑪⑫	7
Zhang et al. (33)	2019	China	Cross-sectional study	≥ 6 months	–	PASP > 35 mmHg	40	40	50.0%	④⑫⑲⑳	7
Li et al. (34)	2019	China	Retrospective cohort study	≥ 3 months	3×/week, 4 h/session	PASP > 35 mmHg	111	72	39.3%	③⑦	9
Liu (35)	2017	China	Retrospective study	≥ 3 months	–	PASP > 35 mmHg	96	100	51.0%	②④⑬⑭⑮	6
He et al. (36)	2017	China	Retrospective study	–	–	PASP > 35 mmHg	65	30	31.6%	⑰㉔	6
Chen et al. (37)	2017	China	Cross-sectional study	≥ 6 months	4 h/session	PASP > 35 mmHg	98	56	36.4%	⑱⑲	7
Zhang (38)	2016	China	Cross-sectional study	≥ 3 months	–	PASP > 35 mmHg	92	36	28.1%	②④⑬⑭⑮	8
Yang et al. (39)	2016	China	Cross-sectional study	≥ 3 months	–	PASP > 35 mmHg	104	59	36.2%	④⑫⑳㉑	8
Wang et al. (40)	2016	China	Cross-sectional study	≥ 3 months	–	PASP > 35 mmHg	86	52	37.7%	④⑯㉑	8
Peng et al. (41)	2016	China	Cross-sectional study	3-6 months	–	PASP > 35 mmHg	64	30	31.9%	①②㉑	8
Yilmaz et al. (42)	2016	Turkey	Prospective study	–	3×/week, 4 h/session	PASP > 35 mmHg	51	26	33.8%	⑤	8
Hsieh et al. (43)	2016	China	Retrospective cohort study	≥ 3 months	3×/week, 4 h/session	PASP > 35 mmHg	109	51	31.9%	④⑧	7
Chen (44)	2015	China	Cross-sectional study	–	–	PASP > 35 mmHg	39	18	31.6%	②⑪⑫⑰	7
He (45)	2015	China	Retrospective study	–	3×/week, 4 h/session	PASP > 35 mmHg	46	52	53.1%	⑬㉒	7
Zhang et al. (46)	2015	China	Cross-sectional study	≥ 6 months	–	PASP > 35 mmHg	56	38	40.4%	㉒	8
Shen et al. (47)	2015	China	Cross-sectional study	≥ 3 months	–	PASP > 35 mmHg	30	30	50.0%	④⑪	8
Fan et al. (48)	2014	China	Cross-sectional study	≥ 3 months	–	PASP > 35 mmHg	38	22	36.7%	④⑳	8
Song et al. (49)	2014	China	Cross-sectional study	≥ 3 months	3×/week, 4 h/session	PASP > 35 mmHg	43	17	28.3%	㉑	8
Bai et al. (50)	2013	China	Retrospective study	–	–	PASP > 35 mmHg	48	36	42.8%	②⑮㉓	8
Pan et al. (51)	2013	China	Cross-sectional study	≥ 6 months	3×/week, 4 h/session	PASP > 40 mmHg	46	36	43.9%	④⑧⑯㉑	8
Pan et al. (52)	2013	China	Cross-sectional study	–	5 sessions/2 weeks; 3.5-4 h/session	PASP > 35 mmHg	187	48	20.4%	㉑	8
Xiao et al. (53)	2013	China	Cross-sectional study	≥ 6 months	3×/week, 4 h/session	PASP > 35 mmHg	79	46	36.8%	⑪⑫㉔	8
Agarwal et al. (54)	2012	America	Prospective cohort study	≥ 3 months	–	PASP > 35 mmHg	178	110	48.2%	④	9
Shen et al. (55)	2011	China	Cross-sectional study	≥ 12 months	3×/week, 4 h/session	PASP > 35 mmHg	66	24	26.7%	㉒㉓	8
Fabbian et al. (56)	2010	Italy	Cross-sectional study	–	–	PASP > 35 mmHg	34	22	39.3%	③	8

Risk factors: ① Alb (albumin); ② Hb (Hemoglobin), ③ Duration of dialysis; ④ LAD (Left atrial diameter); ⑤ Overhydration; ⑥ NT-proBNP (N-terminal pro-brain natriuretic peptide); ⑦ Age; ⑧ Valvular Regurgitation; ⑨ Serous cavity effusion; ⑩ RDW (Red Cell Distribution Width); ⑪ RAD(Right atrial diameter); ⑫ LVEF (Left ventricular ejection fraction); ⑬ BNP (Brain natriuretic peptide); ⑭ hs-CRP (High-sensitivity C-reactive protein); ⑮ E/A (Early diastolic to Atrial contraction velocity ratio); ⑯ LVPWTd (Left Ventricular Posterior Wall Thickness in diastole); ⑰ Interdialytic Weight Gain; ⑱ CD8+; ⑲ CD8+ CD69+; ⑳ LVEDD (Left Ventricular End-Diastolic Diameter); ㉑ LVMI (Left Ventricular Mass Index); ㉒ PTH (Parathyroid Hormone); ㉓ Valvular Calcification; ㉔ Vascular Access Modalities.

PH, pulmonary hypertension; PASP, pulmonary artery systolic pressure.

“–” is not mentioned.

### Prevalence and subgroup analysis of PH in MHD patients

The 39 included studies all provided data for calculating the prevalence rate. The prevalence rate of PH in MHD patients ranged from 9.2% to 56.6%, with an overall prevalence rate of 37% (95% CI: 0.32, 0.42). The summary results are shown in Figure 2. Through the random effects model, it was found that there was a high heterogeneity among the studies (*I*^2^ = 93.6%, < 0.001).

**Figure 2:**
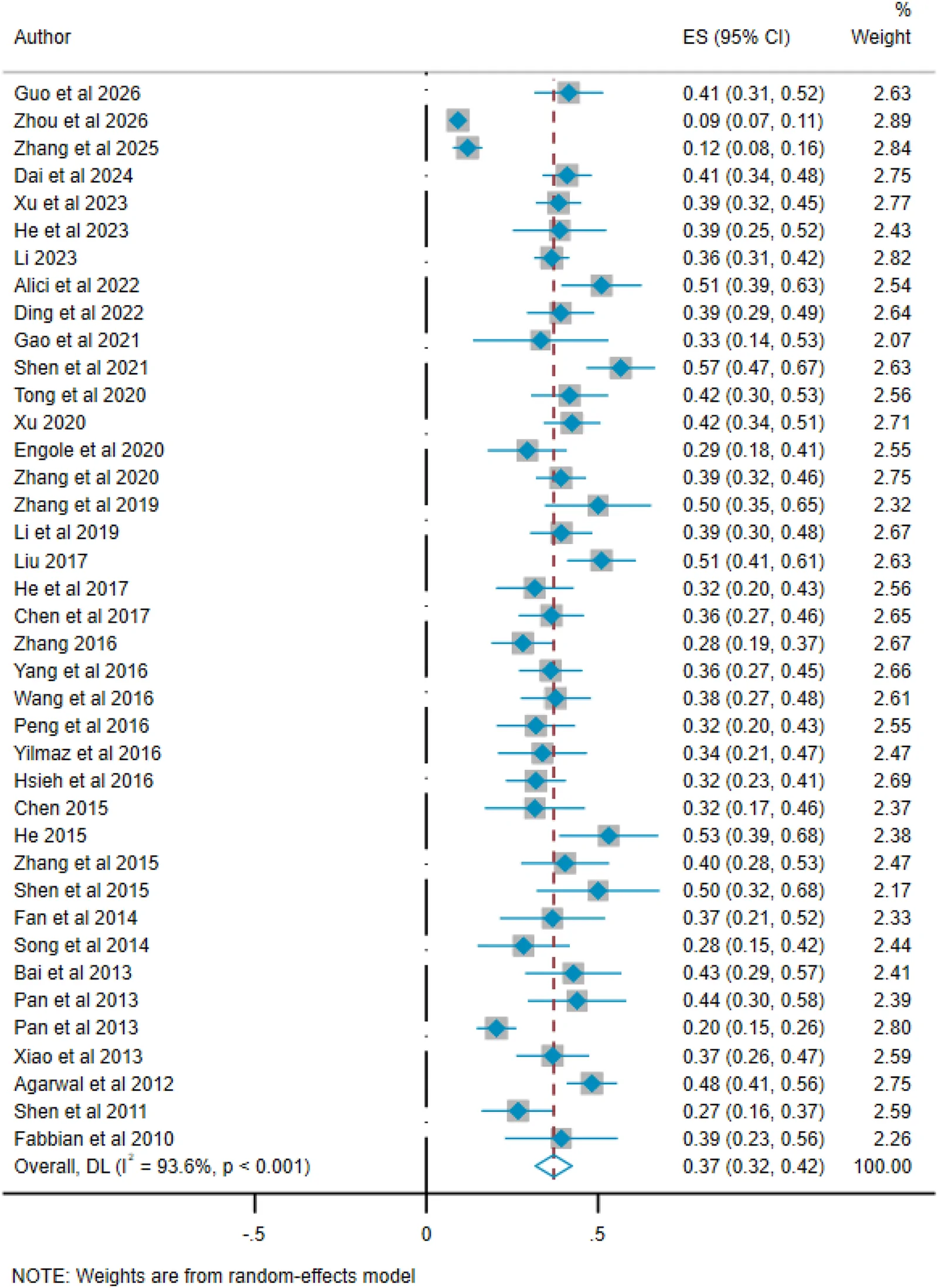
Forest plot of the prevalence of PH in patients with MHD.

To explore potential sources of heterogeneity, subgroup analyses were performed based on country, study design, dialysis duration, and diagnostic criteria for PH. The detailed results are presented in Table 3. Regarding country, the majority of included studies were conducted in China (*n* = 34), yielding a pooled prevalence of 37%. Higher prevalence estimates were observed in Somalia (51%) and the United States (48%), whereas a lower estimate was reported in the Republic of the Congo (29%). With respect to study design, retrospective studies demonstrated the highest prevalence (43%), followed by prospective (42%) and cross-sectional studies (33%). Notably, the cross-sectional study subgroup exhibited substantial heterogeneity (*I*^2^ = 93.3%), indicating that study design may be an important factor affecting the consistency of the results. Concerning dialysis duration, prevalence estimates were comparable between the subgroups of ≥3 months and ≥ 6 months.

**Table 3:** Subgroup analysis of the prevalence of PH in MHD patients.

Subgroups	Country	*N*	Prevalence	95% CI	*I^2^* (%)	*P*
Country	China	34	37%	(0.31, 0.42)	93.8%	< 0.001
	America	1	48%	(0.41, 0.56)	0.0%	< 0.001
	Italy	1	39%	(0.23, 0.56)	0.0%	< 0.001
	Turkey	1	34%	(0.21, 0.47)	0.0%	< 0.001
	Republic of the Congo	1	29%	(0.18, 0.41)	0.0%	< 0.001
	Somalia	1	51%	(0.39, 0.63)	0.0%	< 0.001
Research type	Cross-sectional study	23	33%	(0.27, 0.40)	93.3%	< 0.001
	Retrospective study	12	43%	(0.38, 0.47)	57.7%	0.007
	Prospective study	4	42%	(0.36, 0.48)	33.5%	0.212
Duration of dialysis	≥ 3 months	19	37%	(0.29, 0.46)	96.2%	< 0.001
	≥ 6 months	9	39%	(0.35, 0.42)	0.0%	0.677
	≥ 12 months	1	27%	(0.16, 0.37)	0.0%	< 0.001
	3-6 months	1	32%	(0.20, 0.43)	0.0%	< 0.001
	–	9	36%	(0.35, 0.42)	75.4%	< 0.001
Diagnostic criteria for PH	PASP > 35 mmHg	38	37%	(0.32, 0.42)	93.7%	< 0.001
	PASP > 40 mmHg	1	44%	(0.30, 0.58)	0.0%	< 0.001

MHD, maintenance hemodialysis; PH, pulmonary hypertension; *N*, number of studies; CI, confidence interval; *I*^2^, inconsistency index.

“–” is not mentioned.

### Meta-analysis of associated factors and subgroup analysis results

This meta-analysis identified 13 risk factors and 1 protective factor for PH in MHD patients. These factors were categorized into five domains: general clinical characteristics, laboratory parameters, volume overload-related factors, cardiac function and biomarkers, and echocardiographic structural indices.

This meta-analysis revealed that the following variables were associated with increased risk of PH (all *P* < 0.05): age (OR = 1.03, 95% CI: 1.02–1.05), Vascular Access Modalities (OR = 7.84, 95%CI: 4.98, 12.33), hs-CRP (OR = 3.57, 95%CI: 2.41, 5.30), interdialytic weight gain (continuous [per 1 kg]: OR = 18.39, 95% CI: 3.29–102.69; dichotomous [> 2 kg vs. ≤ 2 kg]: OR = 20.51, 95% CI: 11.04–38.10), serous cavity effusion (OR = 3.03, 95% CI: 1.84–4.97), NT-proBNP (OR = 1.07, 95% CI: 1.02–1.12), BNP (OR = 1.77, 95% CI: 1.06–2.97), E/A (OR = 3.00, 95%CI: 1.89, 4.75), LAD (OR = 1.17, 95%CI: 1.09, 1.25), LVEDD (OR = 1.11, 95% CI: 1.05–1.17), LVPWTd (OR = 1.72, 95% CI: 1.31–2.26), RAD (OR = 1.17, 95% CI: 1.12–1.22), and valvular regurgitation (OR = 3.83, 95% CI: 2.39–6.13). Conversely, LVEF was inversely associated with PH (OR = 0.92, 95% CI: 0.87–0.97).

Substantial heterogeneity was observed for Hb (*I*^2^ = 91.8%). Subgroup analyses stratified by study design, dialysis duration, and quality score failed to adequately explain this heterogeneity. For CD8 + CD69+ (*I*^2^ = 90.0%), subgroup analyses by study type yielded a meaningful reduction in between-study heterogeneity. For BNP (*I*^2^ = 94.2%), we conducted a subgroup analysis by data type, and the results showed that the heterogeneity between studies was reduced to some extent. The unit of measurement may be a source of LAD heterogeneity; nine studies used mm (*I*^2^ = 0.0%), whereas six studies used cm (*I*^2^ = 88.7%). The heterogeneity of RDW and LVEF was high. When studies were removed one by one, the heterogeneity for these two factors decreased, and the results became statistically significant after removal, suggesting that the meta-analysis results may be affected by a single study. Further large-scale studies are needed to validate the influence of these factors on PH development in MHD patients. Detailed results of the meta-analyses and subgroup analyses are presented in Table 4. The detailed results of the meta-analysis and subgroup analyses are presented in Table 4.

**Table 4:** Meta-analysis of associated factors and subgroup-analysis results.

Risk factors	Number of included literature	Sample size (cases)	*I^2^*	*P*	Result		Effect model
					OR (95%CI)	*P*	
General clinical factors							
Age	2	488	0.0%	0.455	1.03 (1.02, 1.05)	**< 0.001**	Fixed effects model
Duration of dialysis	3	398	74.4%	0.020	1.10 (0.94, 1.27)	0.234	Random effects model
Vascular Access Modalities	3	305	38.6%	0.196	7.84 (4.98, 12.33)	**< 0.001**	Fixed effects model
Laboratory test indicators							
Alb	2	253	91.3%	< 0.001	0.27 (0.02, 3.51)	0.316	Random effects model
Hb	8	1081	91.8%	< 0.001	1.03 (0.95, 1.12)	0.429	Random effects model
RDW	3	859	88.6%	< 0.001	1.26 (0.99, 1.61)	0.057	Random effects model
RDW^a^	2	554	20.7%	0.261	1.41 (1.23, 1.61)	< 0.001	Fixed effects model
hs-CRP	2	324	45.9%	0.174	3.57 (2.41, 5.30)	**< 0.001**	Fixed effects model
CD8+	2	281	91.1%	< 0.001	1.14 (0.72, 1.80)	0.576	Random effects model
CD8+ CD69+ (Overall)	3	361	90.0%	< 0.001	0.98 (0.48, 2.01)	0.959	Random effects model
Subgroup analysis by Research type							
Prospective study	1	127	0.0%	< 0.001	2.56 (1.47, 4.47)	0.001	Random effects model
Cross-sectional study	2	234	0.0%	0.995	0.64 (0.51, 0.81)	< 0.001	Random effects model
PTH	3	282	63.3%	0.064	1.003 (1.000, 1.006)	0.081	Random effects model
Capacity load related factors							
Overhydration	2	326	53.4%	0.143	1.90 (0.86, 4.16)	0.111	Random effects model
Interdialytic Weight Gain (Binary classification)	1	95	0.0%	< 0.001	20.51 (11.04, 38.10)	**< 0.001**	Fixed effects model
Interdialytic Weight Gain (Continuous)	2	90	0.0%	0.836	18.39 (3.29, 102.69)	**0.001**	Fixed effects model
Serous cavity effusion	2	448	3.1%	0.356	3.03 (1.84, 4.97)	**< 0.001**	Fixed effects model
Cardiac function and myocardial markers							
NT-proBNP	3	1116	60.9%	0.078	1.07 (1.02, 1.12)	**0.008**	Random effects model
BNP	5	785	94.2%	< 0.001	1.77 (1.06, 2.97)	**0.030**	Random effects model
Subgroup analysis by Data type							
Mean ± SD	3	451	75.8%	0.016	2.41 (1.47, 3.96)	< 0.001	Random effects model
Median	2	334	75.2%	0.045	1.13 (0.83, 1.55)	0.434	Random effects model
E/A	3	408	0.0%	0.678	3.00 (1.89, 4.75)	**< 0.001**	Fixed effects model
Subgroup analysis by Type of variable							
Binary classification	1	84	0.0%	< 0.001	4.04 (1.29, 12.64)	**0.016**	Fixed effects model
Continuous	2	324	0.0%	0.496	2.83 (1.71, 4.68)	**< 0.001**	Fixed effects model
LVEF	8	1312	75.5%	< 0.001	0.92 (0.87, 0.97)	**0.004**	Random effects model
LVEF^b^	6	1092	0.0%	0.468	0.95 (0.93, 0.97)	< 0.001	Fixed effects model
Echocardiographic parameters of cardiac structure							
LAD (Overall)	15	3106	77.0%	< 0.001	1.17 (1.09, 1.25)	**< 0.001**	Random effects model
Subgroup analysis by unit							
mm	9	2111	0.0%	0.457	1.10 (1.07, 1.13)	< 0.001	Random effects model
cm	6	995	88.7%	< 0.001	3.42 (1.51,7.76)	0.003	Random effects model
LVEDD	3	303	0.0%	0.517	1.11 (1.05, 1.17)	**< 0.001**	Fixed effects model
LVMI (Overall)	6	772	82.5%	< 0.001	1.06 (0.97, 1.15)	0.185	Random effects model
Subgroup analysis by sample unit							
g/m^2^	4	374	87.2%	< 0.001	3.21 (1.05, 9.77)	0.040	Random effects model
–	2	398	15.5%	0.277	1.02 (0.92, 1.14)	0.689	Random effects model
LVPWTd	3	376	47.1%	0.151	1.72 (1.31, 2.26)	**< 0.001**	Fixed effects model
RAD	6	973	44.7%	0.093	1.17 (1.12, 1.22)	**< 0.001**	Fixed effects model
Valvular Regurgitation	3	547	0.0%	0.734	3.83 (2.39, 6.13)	**< 0.001**	Fixed effects model
Valvular Calcification	2	174	88.9%	0.003	0.94 (0.008, 10.55)	0.962	Random effects model

*I^2^*, I-squared heterogeneity statistic; OR, odds ratio; CI, confidence interval; Alb, albumin; Hb, hemoglobin; RDW, red cell distribution width; hs-CRP, high-sensitivity C-reactive protein; PTH, parathyroid hormone; NT-proBNP, N-terminal pro-brain natriuretic peptide; BNP, brain natriuretic peptide; E/A, early diastolic to Atrial contraction velocity ratio; LVEF, left ventricular ejection fraction; LAD, left atrial diameter; LVEDD, left ventricular end-diastolic diameter; LVMI, left ventricular mass index; LVPWTd, left ventricular posterior wall thickness in diastole; RAD, right atrial diameter.

Explanations:

1. RDW^a^, The results obtained after excluding the study by Dai et al.

2. LVEF^b^, The results obtained after excluding the study by Yang et al. and Chen.

3. NT-proBNP values reported in ng/L and pg/mL were considered numerically equivalent (1 ng/L = 1 pg/mL); therefore, no unit conversion was applied.

4. Bold font indicates *P* < 0.05.

“–” is not mentioned.

These six predictors—LAD, valvular regurgitation, RAD, LVEF, LVPWTd, and vascular access type—were simultaneously incorporated into both unadjusted and adjusted effect estimates. To evaluate the robustness of the pooled results, we conducted sensitivity analyses for each predictor by re-estimating the pooled effects after excluding studies that provided adjusted data (Hsieh et al., Ding et al., and Engole et al.,). The direction and statistical significance of the pooled effect sizes remained essentially unchanged after exclusion of these studies (see Supplementary File 5), indicating that the pooled results were robust and that the inclusion of adjusted studies did not substantially affect the overall conclusions.

### Meta-regression analysis results

For risk factors examined in 10 or more studies (57), univariate meta-regression was conducted to explore sources of heterogeneity, incorporating study design, dialysis vintage, quality score, sample size, publication year, and PH diagnostic criteria as covariates. Ultimately, only LAD met this threshold (*I*^2^ = 77.0%). The meta-regression revealed that none of these covariates were significantly associated with the effect size (study design: *P* = 0.821; dialysis vintage: *P* = 0.528; quality score: *P* = 0.987; sample size: *P* = 0.470; publication year: *P* = 0.434; PH diagnostic criteria: *P* = 0.244). These findings suggest that the observed heterogeneity may arise from other unaccounted clinical or methodological factors. Full results of the meta-regression are provided in Supplementary File 3.

### Sensitivity analysis results

Sensitivity analyses revealed that the majority of factors produced consistent results under both the fixed-effect and random-effects models, indicating robust conclusions. These factors included age, vascular access modalities, hs-CRP, interdialytic weight gain (Continuous), serous cavity effusion, BNP, NT-proBNP, E/A, LVEF, LAD, LVEDD, LVPWTd, RAD, valvular regurgitation, and valvular calcification. In contrast, several factors exhibited model-dependent effects, with their statistical significance varying between models and thus showing limited robustness. Specifically, Hb, RDW, CD8⁺, CD8⁺CD69⁺, PTH, overhydration, duration of dialysis, Alb, and LVMI were significantly associated with the outcome under the fixed-effect model but lost statistical significance under the random-effects model. Detailed results of the sensitivity analyses are summarized in Table 5.

**Table 5:** Sensitivity analysis results.

Risk factors	Number of studies	Fixed effects model			Random effects model		
		Pooled effect size [OR]	95%CI	*P*	Pooled effect size [OR]	95%CI	*P*
General clinical factors							
Age	2	1.03^a^	(1.02, 1.05)	< 0.001	1.03	(1.02, 1.05)	< 0.001
Duration of dialysis	3	1.03	(1.01, 1.04)	0.010	1.10^a^	(0.94, 1.27)	0.234
Vascular Access Modalities	3	7.84^a^	(4.98, 12.33)	< 0.001	7.40	(4.07, 13.44)	< 0.001
Laboratory test indicators							
Alb	2	0.88	(0.81, 0.97)	0.007	0.27^a^	(0.02, 3.51)	0.316
Hb	8	0.96	(0.95, 0.98)	< 0.001	1.03^a^	(0.95, 1.12)	0.429
RDW	3	1.08	(1.04, 1.12)	< 0.001	1.26^a^	(0.99, 1.61)	0.057
hs-CRP	2	3.57^a^	(2.41, 5.30)	< 0.001	3.73	(2.14, 6.52)	< 0.001
CD8+	2	0.94	(0.89, 0.99)	0.018	1.14^a^	(0.72, 1.80)	0.576
CD8+ CD69+	3	0.79	(0.64, 0.98)	0.034	0.98^a^	(0.48, 2.01)	0.959
PTH	3	1.003	(1.001, 1.005)	< 0.001	1.003^a^	(1.000, 1.006)	0.081
Capacity load related factors							
Overhydration	2	1.55	(1.16, 2.07)	0.003	1.90^a^	(0.86, 4.16)	0.111
Interdialytic Weight Gain (Binary classification)	1	N/A^b^					
Interdialytic Weight Gain (Continuous)	2	18.39^a^	(3.29, 102.69)	0.001	18.39	(3.29, 102.69)	0.001
Serous cavity effusion	2	3.03^a^	(1.84, 4.97)	< 0.001	3.05	(1.83, 5.10)	< 0.001
Cardiac function and myocardial markers							
NT-proBNP	3	1.06	(1.04, 1.08)	< 0.001	1.07^a^	(1.02, 1.12)	0.008
BNP	5	1.001	(1.000, 1.002)	0.046	1.77^a^	(1.06, 2.97)	0.030
E/A	3	3.00^a^	(1.89, 4.75)	< 0.001	3.00	(1.89, 4.75)	< 0.001
LVEF	8	0.94	(0.92, 0.96)	< 0.001	0.92^a^	(0.87, 0.97)	0.004
Echocardiographic parameters of cardiac structure							
LAD	15	1.11	(1.08, 1.14)	< 0.001	1.17^a^	(1.09, 1.25)	< 0.001
LVEDD	3	1.11^a^	(1.05, 1.17)	< 0.001	1.11	(1.05, 1.17)	< 0.001
LVMI	6	1.01	(1.00, 1.01)	0.019	1.06^a^	(0.97, 1.15)	0.185
LVPWTd	3	1.72^a^	(1.31, 2.26)	< 0.001	2.09	(1.22, 3.60)	0.008
RAD	6	1.17^a^	(1.12, 1.22)	< 0.001	1.21	(1.12, 1.30)	< 0.001
Valvular regurgitation	3	3.83^a^	(2.39, 6.13)	< 0.001	3.83	(2.39, 6.13)	< 0.001
Valvular calcification	2	1.00	(0.45, 2.23)	0.994	0.94^a^	(0.008, 10.55)	0.962

CI, confidence interval.

a Main results of the study; Alb, albumin; Hb, hemoglobin; RDW, red cell distribution width; hs-CRP, high-sensitivity C-reactive protein; PTH, parathyroid hormone; NT-proBNP, N-terminal pro-brain natriuretic peptide; BNP, brain natriuretic peptide; E/A, early diastolic to atrial contraction velocity ratio; LVEF, left ventricular ejection fraction; LAD, left atrial diameter; LVEDD, left ventricular end-diastolic diameter; LVMI, left ventricular mass index; LVPWTd, left ventricular posterior wall thickness in diastole; RAD, right atrial diameter.

b Sensitivity analysis was not performed for interdialytic weight gain (binary classification) because only one study reported this variable.

### Grade evaluation of certainty of findings

Evidence quality for all related factors was evaluated using the GRADE system. Given the observational design of all included studies, the initial evidence quality was rated as “low” and was further downgraded due to risk of bias, inconsistency, and imprecision. Ultimately, six related factors were classified as low-quality evidence, while the remaining eighteen were assigned very low-quality evidence (Table 6). The six related factors rated as low-quality evidence were hs-CRP, E/A ratio, LVEDD, LVPWTd, RAD, and valvular regurgitation. This downgrading was primarily attributed to the inherently serious risk of bias in observational studies.

**Table 6:** Summary of findings (SoF) from GRADE assessment of the certainty.

Risk factors	Number of studies	Number of participants (number of PH)	Risk of bias	Inconsistency	Indirectness	Imprecision	Other considerations	OR (95% CI)	Certainty	Importance
Age	2	488	Serious^a^	Not serious	Not serious	Serious^d^	None	1.03 (1.02, 1.05)	⊕○○○ Very low	Important
Duration of dialysis	3	398	Serious^a^	Serious^b^	Not serious	Serious^d^	None	1.10 (0.94, 1.27)	⊕○○○ Very low	Important
Vascular Access Modalities	3	305	Serious^a^	Not serious	Serious^e^	Serious^e^	None	7.84 (4.98, 12.33)	⊕○○○ Very low	Important
Alb	2	253	Serious^a^	Very serious^c^	Not serious	Serious^d^	None	0.27 (0.02, 3.51)	⊕○○○ Very low	Important
Hb	8	1081	Serious^a^	Very serious^c^	Not serious	Serious^d^	None	1.03 (0.95, 1.12)	⊕○○○ Very low	Important
RDW	3	859	Serious^a^	Very serious^c^	Not serious	Serious^d^	None	1.26 (0.99, 1.61)	⊕○○○ Very low	Important
hs-CRP	2	324	Serious^a^	Not serious	Not serious	Not serious	None	3.57 (2.41, 5.30)	⊕⊕○○ Low	Important
CD8+	2	281	Serious^a^	Very serious^c^	Not serious	Serious^d^	None	1.14 (0.72, 1.80)	⊕○○○ Very low	Important
CD8+ CD69+	3	361	Serious^a^	Very serious^c^	Not serious	Serious^d^	None	0.98 (0.48, 2.01)	⊕○○○ Very low	Important
PTH	3	282	Serious^a^	Serious^b^	Not serious	Serious^d^	None	1.003 (1.000 to 1.006)	⊕○○○ Very low	Important
Overhydration	2	326	Serious^a^	Serious^b^	Not serious	Serious^d^	None	1.90 (0.86, 4.16)	⊕○○○ Very low	Important
Interdialytic Weight Gain (Binary classification)	1	95	Serious^a^	Not serious	Not serious	Very Serious^d^	None	20.51 (11.04 to 38.10)	⊕○○○ Very low	Important
Interdialytic Weight Gain (Continuous)	2	90	Serious^a^	Not serious	Not serious	Very Serious^d^	None	18.39 (3.29 to 102.69)	⊕○○○ Very low	Important
Serous cavity effusion	2	448	Serious^a^	Not serious	Not serious	Serious^d^	None	3.03 (1.84, 4.97)	⊕○○○ Very low	Important
NT-proBNP	3	1116	Serious^a^	Serious^b^	Not serious	Not serious	None	1.07 (1.02, 1.12)	⊕○○○ Very low	Critical
BNP	5	785	Serious^a^	Very serious^c^	Not serious	Not serious	None	1.77 (1.06, 2.97)	⊕○○○ Very low	Critical
E/A	3	408	Serious^a^	Not serious	Not serious	Not serious	None	3.00 (1.89, 4.75)	⊕⊕○○ Low	Critical
LVEF	8	1312	Serious^a^	Serious^b^	Not serious	Not serious	None	0.92 (0.87, 0.97)	⊕○○○ Very low	Critical
LAD	15	3106	Serious^a^	Serious^b^	Not serious	Not serious	None	1.17 (1.09, 1.25)	⊕○○○ Very low	Critical
LVEDD	3	303	Serious^a^	Not serious	Not serious	Not serious	None	1.11 (1.05, 1.17)	⊕⊕○○ Low	Critical
LVMI	6	772	Serious^a^	Very serious^c^	Not serious	Serious^d^	None	1.06 (0.97, 1.15)	⊕○○○ Very low	Important
LVPWTd	3	376	Serious^a^	Not serious	Not serious	Not serious	None	1.72 (1.31, 2.26)	⊕⊕○○ Low	Critical
RAD	6	973	Serious^a^	Not serious	Not serious	Not serious	None	1.17 (1.12, 1.22)	⊕⊕○○ Low	Critical
Valvular Regurgitation	3	547	Serious^a^	Not serious	Not serious	Not serious	None	3.83 (2.39, 6.13)	⊕⊕○○ Low	Critical
Valvular Calcification	2	174	Serious^a^	Very serious^c^	Not serious	Serious^d^	None	0.94 (0.008, 10.55)	⊕○○○ Very low	Important

GRADE, Grading of Recommendations Assessment, Development and Evaluation; PH, pulmonary hypertension; CI, confidence interval; OR, odds ratio; Alb, albumin; Hb, hemoglobin; RDW, red cell distribution width; hs-CRP, high-sensitivity C-reactive protein; PTH, parathyroid hormone; NT-proBNP, N-terminal pro-brain natriuretic peptide; BNP, brain natriuretic peptide; E/A, early diastolic to atrial contraction velocity ratio; LVEF, left ventricular ejection fraction; LAD, left atrial diameter; LVEDD, left ventricular end-diastolic diameter; LVMI, left ventricular mass index; LVPWTd, left ventricular posterior wall thickness in diastole; RAD, right atrial diameter.

Explanations:.

a All included studies were observational design. Although most of them had high NOS scores (≥ 7), the risk of bias was rated as Serious due to differences in exposure measurement, residual confounding and information bias in some retrospective data.

b Downgraded due to high heterogeneity caused by *I*^2^ > 50%.

c Downgraded due to significant heterogeneity caused by *I*^2^ > 70%.

d Confidence intervals are wide, the sample size is small, or the sample crosses the null line.

e Downgrade due to mixed definitions of variables (continuous vs. dichotomous) across studies.

Importance assessed based on clinical relevance, modifiability, and strength of association with PH outcomes in MHD patients.

The remaining eighteen related factors—age, duration of dialysis, vascular access modalities, albumin, hemoglobin, RDW, CD8⁺, CD8⁺CD69⁺, PTH, overhydration, interdialytic weight gain (binary classification and continuous), serous cavity effusion, NT-proBNP, BNP, LVEF, LAD, LVMI, and valvular calcification—were all graded as very low-quality evidence. Reasons for further downgrading included serious inconsistency (*I*^2^ > 50% or > 70%) and serious imprecision (small sample size, wide confidence intervals, or crossing the line of no effect). Notably, although LAD had the largest number of included studies (fifteen studies; 3,106 patients), its evidence quality remained very low because of substantial heterogeneity (*I*^2^ = 77.0%) that could not be fully resolved through meta-regression.

In summary, the overall evidence quality regarding related factors for PH in MHD patients is low. Although the direction of association was consistent across most studies, the true effect size may differ substantially from the pooled estimates, and clinical interpretation should be made with caution. High-quality prospective cohort studies are needed to confirm these findings.

### Results of publication bias analysis

Publication bias was assessed using Egger's regression test for the pooled prevalence and for risk factors with ≥ 10 included studies. For the overall prevalence, Egger's test revealed a statistically significant small-study effect (intercept = 5.836, 95% CI: 4.421–7.252, *P* *<* *0.001*), indicating the presence of publication bias. The slope coefficient was also significant (coefficient = 0.067, 95% CI: 0.012–0.122, *P* *=* *0.018*). As shown in Figure 3, the scatterplot exhibited an overall upward trend with increasing precision, accompanied by an asymmetric distribution and a high-precision outlier on the right side. This pattern suggests that small-sample studies may have underestimated the true effect size or that studies reporting null or negative findings may have remained unpublished.

**Figure 3:**
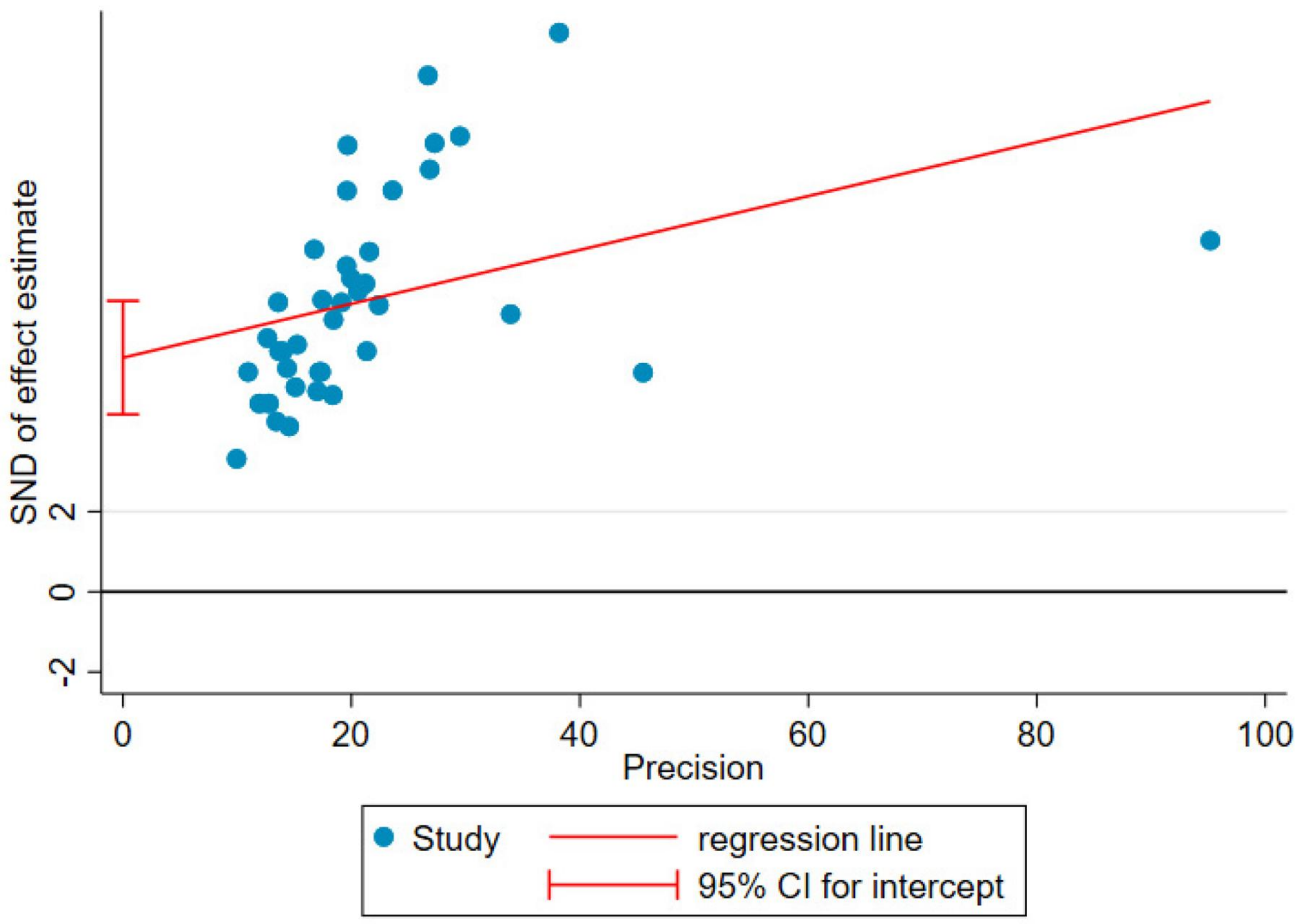
Egger's test of prevalence rate.

Among the associated factors examined, only LAD met the inclusion criterion of ≥ 10 studies. Egger's test for LAD likewise demonstrated a significant small-study effect (intercept = 3.036, 95% CI: 2.255–3.817, *P* *<* *0.001*), whereas the slope test did not reach statistical significance (coefficient = −0.018, 95% CI: −0.057–0.021, *P* *=* *0.334*). As illustrated in Figure 4, studies with lower precision were predominantly distributed above the regression line, resulting in an asymmetric funnel plot that suggests possible overestimation of the LAD effect size in smaller studies.

**Figure 4:**
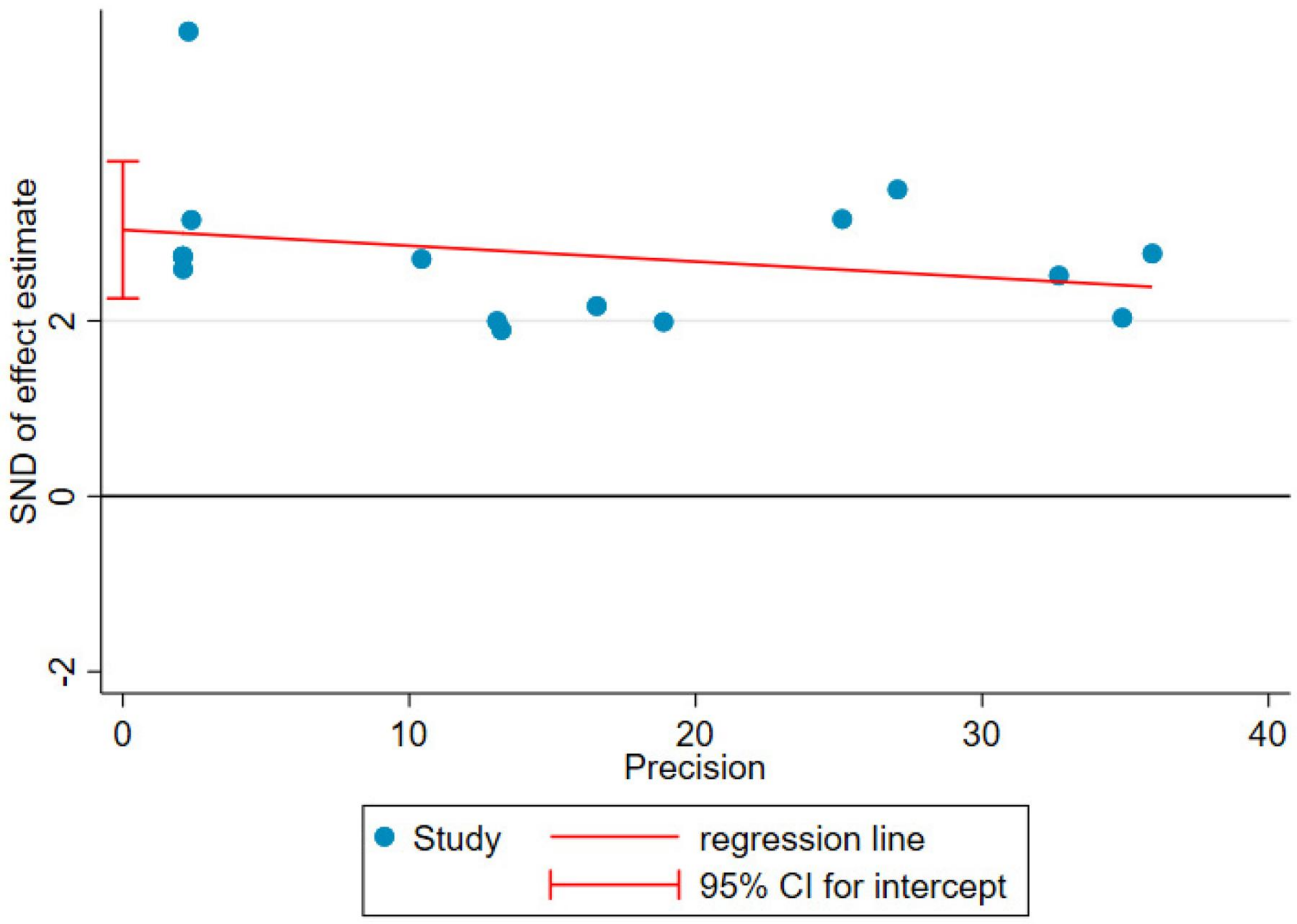
Egger's test of LAD.

In summary, publication bias was detected in both the prevalence meta-analysis and the LAD subgroup analysis. Therefore, caution is warranted when interpreting the pooled effect estimates, as the observed associations may be somewhat inflated.

## Discussion

This systematic review and meta-analysis aimed to estimate the overall prevalence of PH and identify its associated risk factors in patients undergoing MHD. The pooled prevalence was 37%, which aligns with the findings reported by Noah C. Schoenberg et al. (58). But it is lower than the 42% reported in a previous meta-analysis by Lin et al. (7). This discrepancy may be attributed to differences in study inclusion criteria.

Existing evidence indicates that PH adversely affects the prognosis of patients on MHD, not only reducing quality of life but also significantly shortening overall survival. For instance, a systematic review and meta-analysis of 16 studies involving 7,112 patients demonstrated that ESRD patients with PH on maintenance dialysis had a 2.32-fold higher risk of all-cause mortality (3). Similarly, a prospective study by Ramasubbu K et al. showed that among chronic hemodialysis patients with PH, mortality was 26% in those with PH and 6% in those without PH (59). These findings underscore the importance of identifying risk factors for PH in MHD patients, which is crucial for implementing targeted interventions aimed at mitigating both its incidence and associated mortality.

Regarding general clinical factors, both advanced age and the type of vascular access modality were associated with PH in MHD patients (Certainty of evidence: very low). In the two studies reporting age, patients with PH were all aged 60 years or older. The meta-analysis indicated that each one-year increase in age was associated with an approximately 3% higher risk of PH. The underlying mechanism may involve age-related dysfunction in pulmonary vascular endothelial cells and smooth muscle cells (60). A study (61) from India showed that the incidence of PH in patients with arteriovenous fistula (AVF) was as high as 62.5%, while it ranged from 5% to 20% in patients with a temporary catheter. All three included studies suggested that AVF as a vascular access mode may increase the risk of PH. However, it is important to note that because these three studies reported unadjusted ORs, we cannot rule out residual confounding factors, such as dialysis age, comorbidity burden, and disease severity. Even with a combined effect size of 7.84, this estimate reflects only the strength of the association between the variables and cannot be considered an adjusted effect size; the true magnitude of the association may be distorted by unmeasured confounding. Previous studies (9, 56) found that longer dialysis vintage was associated with a higher risk of PH. However, this meta-analysis did not confirm this association (OR = 1.10, 95% CI: 0.94–1.27, *P* *=* *0.234*), likely due to only 3 of the 39 included studies reporting this factor using inconsistent definitions. Future prospective studies with standardized assessment are warranted.

According to the Laboratory test indicators, seven risk factors, including Alb, Hb, RDW, hs-CRP, CD8+, CD8 + CD69+, and PTH, were initially screened in this study. Ultimately, only hs-CRP was associated with an increased risk of PH (certainty of evidence: low). Previous studies have demonstrated that micro-inflammation is common in MHD, and this chronic low-grade inflammation is closely associated with atherosclerosis and various metabolic disorders (62). hs-CRP can stimulate monocytes to secrete IL-6 and TNF-*α*, promote the production of ICAM-1, VCAM-1, and other adhesion molecules, trigger inflammatory responses, and cause vascular endothelial damage (63, 64). hs-CRP is not only involved in the pathogenesis of PH, but can also serve as a biomarker to predict the severity and treatment response of PH (65).

Interdialytic weight gain serves as a direct reflection of the severity of sodium and water retention between dialysis sessions. Studies have shown that an IDWG exceeding 3.5% of dry body weight can increase pulmonary artery pressure and exacerbate pulmonary congestion (66). Therefore, rigorous volume management is central to preventing PH in MHD patients. Clinical strategies should prioritize sodium restriction, recommending a daily sodium intake of less than 2 grams. Furthermore, the ultrafiltration volume per session should not exceed 3% of dry body weight, and dry weight should be reassessed at least monthly (67). Notably, only 3 studies (*n* = 185) included in this meta-analysis reported IDWG data, and definitions of IDWG varied across studies: 2 studies analyzed it as a continuous variable (each additional 1 kg), and 1 study used a dichotomous cutoff value (> 2 kg vs. ≤ 2 kg). Because these effect estimates are based on different exposure scales, they were not pooled into a single overall estimate. Instead, they are reported separately: the pooled OR for the continuous-variable subgroup was 18.39 (95% CI: 3.29–102.69), and the OR for the dichotomous variable subgroup was 20.51 (95% CI: 11.04–38.10). Both analyses indicate a consistent positive association between greater IDWG and PH risk. However, the limited number of included studies, inconsistent variable definitions, and the wide confidence interval for the continuous-variable subgroup preclude a precise estimate of the effect size. These findings should therefore be interpreted with caution (certainty of evidence: very low). Such effusions (e.g., pleural, peritoneal, or pericardial) represent a clinical consequence of chronic volume overload. Their presence not only signifies severe systemic fluid retention but also mechanically increases intrathoracic pressure—thereby limiting pulmonary vascular distension, reducing lung compliance, and further elevating pulmonary vascular resistance (68, 69).

In cardiac function and myocardial markers, NT-proBNP, BNP, and the E/A velocity ratio were positively associated with PH in MHD patients, while LVEF showed an inverse association (OR = 0.92, *P* *<* *0.004*). The certainty of evidence for these associations was rated as very low for NT-proBNP and BNP, and low for the E/A velocity ratio, and low for BNP. The pooled OR for NT-proBNP was 1.07, indicating that higher NT-proBNP levels were associated with increased odds of PH. This finding aligns with previous studies (70, 71). NT-proBNP is recommended by international guidelines as a key biomarker for the diagnosis, risk stratification, and prognosis of PH (72). Notably, the relationship between E/A ratio and PH may not be strictly linear. Emerging evidence suggests that both reduced E/A ratios (< 1, indicating impaired relaxation) and elevated E/A ratios (> 2, suggesting restrictive filling) are associated with an increased risk of PH (73), implying a potential U-shaped association. However, since the primary studies included in our meta-analysis reported E/A predominantly as a continuous variable and did not provide category-specific data, we were unable to conduct a threshold-effect analysis. Future studies should report E/A ratios using clinically meaningful categories to clarify this non-linear relationship. LVEF was identified as a protective factor (OR = 0.92, *P* *<* *0.004*), suggesting that MHD patients with preserved systolic function may have a lower likelihood of developing PH. A study involving 475 dialysis patients demonstrated that each 5% increase in LVEF was associated with an 8% reduction in PH-related mortality risk (74). These findings underscore the importance of monitoring and preserving cardiac systolic function in MHD patients.

Multiple echocardiographic structural parameters were closely associated with PH development in MHD patients. Meta-analysis demonstrated that each unit increase in LAD was associated with a 17% higher odds of PH (OR = 1.17, *P* *<* *0.001*), although the certainty of evidence was rated as very low. Left atrial enlargement typically reflects chronically elevated left atrial pressure secondary to impaired left ventricular diastolic function. Retrograde transmission of elevated left atrial pressure to the pulmonary veins elevates pulmonary capillary wedge pressure, thereby precipitating or exacerbating PH (75). Additionally, enlarged LVEDD and thickened LVPWTd were both associated with an increased risk of PH (certainty of evidence: low). An increased LVEDD indicates left ventricular volume overload, whereas increased LVPWTd suggests pressure overload with concentric left ventricular remodeling. Both conditions impair left ventricular diastolic function, leading to elevated left atrial pressure and consequent pulmonary circulation pressure (76).

Regarding right heart structural changes and valvular lesions, RAD enlargement was also associated with an increased risk of PH (OR: 1.17), with low certainty of evidence—a finding consistent with prior research (77, 78). Valvular regurgitation is highly prevalent among MHD patients and is associated with uremic cardiomyopathy, disturbances in calcium-phosphorus metabolism, and valvular calcification (79). The present meta-analysis demonstrated that valvular regurgitation was associated with nearly a threefold increased risk of PH (OR: 3.83), with low certainty of evidence. Mitral and tricuspid regurgitation further aggravate cardiac volume overload, leading to cardiac chamber dilation, worsening pulmonary congestion, and a direct elevation in pulmonary circulation pressure, thereby accelerating both the onset and progression of PH (52). The cumulative interplay of these risk factors may be further associated with an increased likelihood of PH in MHD patients.

Regional concentration of included studies. The majority of studies in our meta-analysis originated from China (87.2%), which represents both a strength and a limitation of this work. This geographic concentration ensures relative homogeneity in dialysis protocols and patient management practices across the included studies, thereby reducing clinical heterogeneity. Conversely, this concentration introduces notable regional and ethnic selection bias. China has one of the largest ESRD populations worldwide and a broadly accessible maintenance hemodialysis system, which naturally yields a high volume of related research outputs (80). However, the scarcity of relevant evidence from Western countries, South Asia, Africa, and Latin America means that our findings may not fully capture global variations in the epidemiological characteristics of PH among MHD patients. Therefore, clinicians and policymakers outside China should interpret our pooled estimates as hypothesis-generating rather than definitive. We strongly encourage investigators in underrepresented regions to conduct similar studies to enrich the global evidence base.

### Strengths and limitations

The primary strength of this review is its rigorous methodology, which strictly adhered to PRISMA 2020 guidelines and was prospectively registered in PROSPERO. Furthermore, by focusing specifically on patients receiving MHD—rather than the broader chronic kidney disease population examined in prior syntheses—this review provides more targeted insights into the unique factors influencing PH development in the dialysis setting. However, several limitations warrant consideration. All included studies were observational, and a substantial proportion employed cross-sectional or retrospective designs, limiting causal inference and leaving residual confounding unaddressed. Notably, 34 of 39 studies (87.2%) were conducted in China, with only five from other countries (the United States, Italy, Turkey, Somalia, and the Republic of the Congo). While this distribution reflects China's large MHD population and high research output, it introduces substantial geographic and ethnic selection bias. Consequently, our pooled estimates should be generalized to non-Asian populations or different healthcare systems with considerable caution. Heterogeneity in diagnostic criteria for PH may have affected the consistency of prevalence estimates. For several risk factors, the meta-analysis included a limited number of studies with high heterogeneity. Moreover, the GRADE assessment indicated that the certainty of evidence for most factors was low or very low, implying that their clinical application requires careful judgment. A major limitation of this meta-analysis is the scarcity of adjusted effect estimates among the included studies: only 3 of 39 (7.7%) reported adjusted ORs, while the remaining 36 (92.3%) reported only unadjusted ORs, potentially leaving the combined estimates susceptible to residual confounding. Therefore, the findings should be interpreted as associational rather than causal. Future primary studies should report fully adjusted estimates using standardized covariate sets to enable more robust meta-analytic synthesis. Additionally, the included studies varied in ESKD etiologies (e.g., diabetic nephropathy, glomerulonephritis) and in the reporting of comorbidities such as diabetes, hypertension, and cardiovascular disease. These factors represent potential sources of heterogeneity; however, corresponding subgroup analyses were precluded by insufficient data from primary studies.

## Conclusion

In conclusion, this meta-analysis estimates the pooled prevalence of PH among MHD patients at 37%. Several potentially relevant factors were identified, including advanced age, arteriovenous fistula use, elevated inflammatory markers, interdialytic weight gain (binary classification and continuous), serous cavity effusion, and abnormal or structural cardiac changes, whereas left ventricular ejection fraction showed an inverse association with PH. However, the evidence base consists predominantly of observational studies with low overall certainty and substantial heterogeneity, limiting causal inference and the precision of effect estimates. Future research should prioritize prospective cohort studies with standardized PH diagnostic criteria and adequate sample sizes; establishing multinational MHD registries with uniform screening protocols would further support rigorous prospective investigations. Such initiatives are essential to inform early identification and personalized management of this high-risk population.

## Data Availability

The original contributions presented in the study are included in the article/Supplementary Material, further inquiries can be directed to the corresponding author.

## Supplementary material

Supplementary File 1 PRISMA-ScR checklist.

Supplementary File 2 Search strategies.

Supplementary File 3 Meta-regression results.

SUPPLEMENTARY File 4 Publication bias.

Supplementary File 5 Metaninf analysis.
