# Supplementary material for: Prevalence and factors associated with pulmonary hypertension in patients undergoing maintenance hemodialysis: a systematic review and meta-analysis
Source: Front Med (Lausanne). 2026 Sep 28;13:1889032. doi: 10.3389/fmed.2026.1889032 (PMC13658899; doi:10.3389/fmed.2026.1889032)
Supplement: Supplementary file 2 [file Table2.docx]

**CNKI, VIP**

(Topic: renal dialysis + dialysis + renal replacement therapy + renal replacement + hemodialysis + hemodiafiltration + hemodialysis + extracorporeal dialysis) AND (Topic: pulmonary hypertension + pulmonary artery, hypertension + pulmonary hypertension) AND (Topic: Influencing factors + related factors + factors + risk factors + risk factors + predictive factors + influencing factors + risk factors)

**Wan Fang, CBM, Chinese Medical Association**

(Topic: Renal dialysis OR dialysis OR renal replacement therapy OR renal replacement OR hemodialysis OR hemodiafiltration OR hemodialysis OR extracorporeal dialysis) AND (Topic: Pulmonary arterial hypertension OR pulmonary arterial hypertension, hypertension OR pulmonary arterial hypertension) AND (Topic: Influencing factors OR related factors OR factors OR risk factors OR risk factors OR predictive factors OR influencing factors OR risk factors

PubMed

#1 (((((((((Renal Dialysis[MeSH Terms]) OR (Dialyses, Renal[Title/Abstract])) OR (Renal Dialyses[Title/Abstract])) OR (Dialysis, Renal[Title/Abstract])) OR (Hemodialysis[Title/Abstract])) OR (Hemodialyses[Title/Abstract])) OR (Dialysis, Extracorporeal[Title/Abstract])) OR (Dialyses, Extracorporeal[Title/Abstract])) OR (Extracorporeal Dialyses[Title/Abstract])) OR (Extracorporeal Dialysis[Title/Abstract])

#2 (((Pulmonary Arterial Hypertension[MeSH Terms]) OR (Pulmonary Arterial Hypertension[Title/Abstract])) OR (Arterial Hypertension, Pulmonary[Title/Abstract])) OR (Hypertension, Pulmonary Arterial[Title/Abstract])

#3 #1 AND ##2

**Cochrane Library**

#1	MeSH descriptor: [Renal Dialysis] explode all trees	7595

#2	(Renal Dialysis):ti,ab,kw OR (Dialyses, Renal):ti,ab,kw OR (Renal Dialyses):ti,ab,kw OR (Dialysis, Renal):ti,ab,kw OR (Hemodialysis):ti,ab,kw	21585

#3	(Hemodialyses):ti,ab,kw OR (Dialysis, Extracorporeal):ti,ab,kw OR (Dialyses, Extracorporeal):ti,ab,kw OR (Extracorporeal Dialyses):ti,ab,kw OR (Extracorporeal Dialysis):ti,ab,kw	397

#4	MeSH descriptor: [Pulmonary Arterial Hypertension] explode all trees	249

#5	(Pulmonary Arterial Hypertension):ti,ab,kw OR (Arterial Hypertension, Pulmonary):ti,ab,kw OR (Hypertension, Pulmonary Arterial):ti,ab,kw	3074

#6	(Influencing factors):ti,ab,kw OR (related factors):ti,ab,kw OR (factors):ti,ab,kw OR (predictors factors):ti,ab,kw	261652

#7	#1 OR #2 OR #3	22266

#8	#4 OR #5	3074

#9	#7 AND #8	29

WOS

#1 (((((((((TS=(Renal Dialysis)) OR TS=(Dialyses, Renal)) OR TS=(Renal Dialyses)) OR TS=(Dialysis, Renal)) OR TS=(Hemodialysis)) OR TS=(Hemodialyses)) OR TS=(Dialysis, Extracorporeal)) OR TS=(Dialyses, Extracorporeal)) OR TS=(Extracorporeal Dialyses)) OR TS=(Extracorporeal Dialysis)

#2 ((TS=(Pulmonary Arterial Hypertension)) OR TS=(Arterial Hypertension, Pulmonary)) OR TS=(Hypertension, Pulmonary Arterial)

#3 (((TS=(Influencing factors)) OR TS=(related factors)) OR TS=(factors)) OR TS=(predictors factors)

#4 #1 AND #2 AND #3
