# Supplementary material for: Prevalence and factors associated with pulmonary hypertension in patients undergoing maintenance hemodialysis: a systematic review and meta-analysis
Source: Front Med (Lausanne). 2026 Sep 28;13:1889032. doi: 10.3389/fmed.2026.1889032 (PMC13658899; doi:10.3389/fmed.2026.1889032)
Supplement: Supplementary file 4 [file Table4.docx]

**LAD**

**Valvular Regurgitation**

**RAD**

**LVEF**

**LVPWTd**

**Vascular Access Modalities**

| Variable | All included in OR (95% CI) | Excluding adjusted OR (95%CI) | Object of exclusion |
| --- | --- | --- | --- |
| LAD | 1.17 (1.09, 1.25) | 1.19 (1.10, 1.28) | Hsieh et al |
| Valvular Regurgitation | 3.83 (2.39, 6.13) | 3.87 (2.17, 6.91) | Hsieh et al |
| RAD | 1.17 (1.12, 1.22) | 1.17 (1.12, 1.22) | Ding et al |
| LVEF | 0.92 (0.87, 0.97) | 0.94 (0.92, 0.97) | Ding et al |
| LVPWTd | 1.72 (1.31, 2.26) | 3.11 (1.61, 6.01) | Ding et al |
| Vascular Access Modalities | 7.84 (4.98, 12.33) | 9.67 (5.75, 16.27) | Engole et al |
