# Supplementary material for: Prevalence and factors associated with pulmonary hypertension in patients undergoing maintenance hemodialysis: a systematic review and meta-analysis
Source: Front Med (Lausanne). 2026 Sep 28;13:1889032. doi: 10.3389/fmed.2026.1889032 (PMC13658899; doi:10.3389/fmed.2026.1889032)
Supplement: Supplementary file 5 [file Table5.docx]

Meta regression

**表3 单变量Meta回归分析结果
Table 3 Results of univariate meta-regression analysis**

| 协变量 Covariate | 系数 Coefficient | 标准误 Std. Err. | t值 t | P值 P | 95% CI | τ² | I²_res (%) | Adj R² (%) |
| --- | --- | --- | --- | --- | --- | --- | --- | --- |
| 研究设计（回顾性+前瞻性队列 vs 横断面） Study design (Retrospective + Prospective cohort vs Cross-sectional) | -0.0155 | 0.0672 | -0.23 | 0.821 | -0.161 ~ 0.130 | 0.000493 | 79.12 | -68.02 |
| 透析持续时间（月） Duration of dialysis (months) | 0.0275 | 0.0423 | 0.65 | 0.528 | -0.064 ~ 0.119 | 0 | 78.61 | 100.00 |
| 质量评分 Quality score | -0.0011 | 0.0635 | -0.02 | 0.987 | -0.138 ~ 0.136 | 0.000645 | 79.27 | -119.86 |
| 样本量 Sample size | 0.0491 | 0.0660 | 0.74 | 0.470 | -0.094 ~ 0.192 | 0 | 78.40 | 100.00 |
| 发表年份 Publication year | 0.0074 | 0.0092 | 0.81 | 0.434 | -0.012 ~ 0.027 | 0.000308 | 78.18 | -4.94 |
| PAH诊断标准 PAH diagnostic criteria | 0.6014 | 0.4932 | 1.22 | 0.244 | -0.464 ~ 1.667 | 0.000202 | 76.94 | 30.97 |

注：所有Meta回归均采用限制性最大似然法（ReML）估计研究间方差，并使用Knapp-Hartung校正计算置信区间。

Note: All meta-regressions used restricted maximum likelihood (ReML) for between-study variance estimation with Knapp-Hartung adjustment for confidence intervals.

a 研究设计分类：类型1=横断面研究（n=10），类型2=回顾性研究（n=4），类型3=前瞻性队列研究（n=1）。由于前瞻性队列研究仅1项（Agarwal et al），故将类型2与类型3合并后与类型1（横断面研究，参照组）比较。

a Study design classification: Type 1 = cross-sectional studies (n=10), Type 2 = retrospective studies (n=4), Type 3 = prospective cohort studies (n=1). Since prospective cohort studies comprised only 1 study (Agarwal et al), Type 2 and Type 3 were merged for comparison with Type 1 (cross-sectional studies, reference group).

b τ²=0 为边界解（boundary solution），不代表异质性完全消除，系小样本Meta回归中的常见数值现象。

b τ²=0 represents a boundary solution and does not indicate complete elimination of heterogeneity; this is a common numerical phenomenon in small-sample meta-regression.
