# Supplementary material for: Prevalence and factors associated with pulmonary hypertension in patients undergoing maintenance hemodialysis: a systematic review and meta-analysis
Source: Front Med (Lausanne). 2026 Sep 28;13:1889032. doi: 10.3389/fmed.2026.1889032 (PMC13658899; doi:10.3389/fmed.2026.1889032)
Supplement: Supplementary file 6 [file Table3.docx]

**Results of publication bias analysis**

1. **prevalence rate**

2. **LAD**
